# Call for Role Development and Application of the Monitoring Profile in ADEs and ADRs

**DOI:** 10.3390/pharmacy6040118

**Published:** 2018-10-25

**Authors:** Mojtaba Vaismoradi

**Affiliations:** Faculty of Nursing and Health Sciences, Nord University, 8049 Bodø, Norway; mojtaba.vaismoradi@nord.no

Adverse Drug Events (ADEs) are injuries resulting from medicine-related interventions. They encompass medication errors, Adverse Drug Reactions (ADRs), and allergic reactions, etc. As a matter of clarification, an ADE is a harm associated with any dose of medication, but an ADR is a harm caused by a medication dose used commonly in prescription. Therefore, an ADR can be considered a subtype of an ADE [1]. It is believed that ADEs may happen in hospitals, long-term care settings, and outpatient clinics, but they are mainly preventable. Reducing the number of ADEs can improve the quality and safety of healthcare systems, reduce healthcare costs and readmission rates, and lead to higher satisfaction with healthcare services [2]. Therefore, preventive and pro-active interventions on medicine prescribing, dispensing, administrating, and sufficient monitoring and reporting of ADEs have been emphasized by international organizations. For instance, the World Health Organization (WHO) in 2017 launched a global initiative to reduce avoidable medicine-associated harm by 50% over the next five years across the globe. The WHO has also called all countries to devise priority interventions for addressing the improper use of medicines with a high risk of harm, use of multiple medicines by patients suffering from various health-related conditions, and transition of care in the healthcare system [3]. In this respect, sensitizing the public with regard to the significance of ADEs and developing evidence-based guidelines to ensure the monitoring of medicines are required. Also, sharing knowledge and improving collaboration between multi-professional healthcare providers with various geographic settings and languages can also help with this aim [4].

In line with the above-mentioned aim, the Special issue of “*Patient Safety and Adverse Drug Events in Medication Practice*” opened the discussion on medicine management and called for article submissions by international and multi-professional researchers. The rigorous peer-review process managed by the journal’s editorial office led to the acceptance and publication of five articles authored by researchers from Australia, India, Iran, Ireland, Malaysia, Norway, Pakistan, Saudi Arabia, and the United Kingdom.

Briefly, they have developed knowledge on the healthcare providers’ role in pharmacovigilance, barriers to the reporting and monitoring of ADEs/ADRs [5,6,7], *pro re nata* (PRN) as medicines administration based on patients’ needs and related ADEs [8], and initiatives for the systematic monitoring of ADEs/ADRs [9]. These studies have provided worthwhile findings with many implications for education, practice, policy making, and future research. As concerns shared by these studies, there is a need to develop the healthcare providers’ role and facilitate the reporting and devising of monitoring systems of ADEs/ADRs.

For role development, besides physicians’ and pharmacists’ involvement in pharmacovigilance activities [5,6,7], it is advised that nurses become central actors in medicine surveillance. Nurses always have had a critical role in patient safety through their constant presence at the bedside and collaboration with other healthcare providers and family caregivers [8,10,11]. Therefore, they are able to detect medication issues that may be overlooked by other healthcare providers, and they can report them to ensure patient safety. 

For the reporting and monitoring of ADEs/ADRs, for instance, the American Society of Health-System Pharmacists (ASHP) has published some guidelines on ADRs surveillance, monitoring, and reporting. It has asked that any ADRs program should incorporate evaluating, documenting, and reporting of ADRs as well as providing feedbacks to healthcare professionals and patients, with the aim of creating a positive change in medicine management [12]. Another example is the medicine monitoring program, entitled the Adverse Drug Reaction Profile (ADRe), that has been introduced in one the articles published in this Special Issue. The ADRe is on the basis of collaboration by nursing professionals for multidisciplinary interventions. It provides nurses with a structured method for medicine monitoring to identify ADRs with the aim of improving the health and wellbeing of patients. Therefore, nurses complete the profile and pass highlighted problems to pharmacists and physicians to inform a medication review. The multidisciplinary team evaluates the causes of problems identified, and whether there is a need to change medicines [9,13].

In conclusion, the application of these on-going monitoring profiles in other settings requires well-designed studies and the context-based adaptation of these profiles. Further studies are needed to assess ADEs/ADRs monitoring profiles in terms of benefits taken by healthcare organizations and healthcare professionals, identification of their weaknesses and strengths, incorporation of monitoring profiles into risk-management initiatives, and their effects on the quality of healthcare services.

## References

[1] National Coordinating Council for Medication Error Reporting and Prevention (NCCMERP) (2015). Contemporary View of Medication–Related Harm A New Paradigm. https://www.nccmerp.org/sites/default/files/nccmerp_fact_sheet_2015-02-v91.pdf.

[2] Office of Disease Prevention and Health Promotion (ODPHP) (2018). Overview: Adverse Drug Events. https://health.gov/hcq/ade.asp.

[3] World Health Organization (WHO) (2017). WHO Launches Global Effort to Halve Medication-Related Errors in 5 Years. http://www.who.int/mediacentre/news/releases/2017/medication-related-errors/en/.

[4] International Council of Nurses (ICN) (2018). Patient Safety Global Ministerial Summit 2018, International Council of Nurses (ICN) Statement. https://www.icn.ch/sites/default/files/inline-files/Glob%20Minist.%20Pt%20Safety%20Summit_ICN%20statement%20final%20%281%29.pdf.

[5] Sundaran S., Udayan A., Hareendranath K., Eliyas B., Ganesan B., Hassan A., Subash R., Palakkal V., Salahudeen M.S. (2018). Study on the Classification, Causality, Preventability and Severity of Adverse Drug Reaction Using Spontaneous Reporting System in Hospitalized Patients. Pharmacy.

[6] Syed A., Azhar S., Raza M.M., Saeed H., Jamshed S.Q. (2018). Assessment of Knowledge, Attitude and Barriers towards Pharmacovigilance among Physicians and Pharmacists of Abbottabad, Pakistan. Pharmacy.

[7] Barrett M., Keating A., Lynch D., Scanlon G., Kigathi M., Corcoran F., Sahm L.J. (2018). Clozapine Patients at the Interface between Primary and Secondary Care. Pharmacy.

[8] Vaismoradi M., Amaniyan S., Jordan S. (2018). Patient Safety and Pro Re Nata Prescription and Administration: A Systematic Review. Pharmacy.

[9] Jordan S., Logan P.A., Panes G., Vaismoradi M., Hughes D. (2018). Adverse Drug Reactions, Power, Harm Reduction, Regulation and the ADRe Profiles. Pharmacy.

[10] Agency for Healthcare Research and Quality (AHRQ) Patient Safety Network (PSNet). Nursing and Patient Safety. https://psnet.ahrq.gov/primers/primer/22/Nursing-and-Patient-Safety.

[11] Bigi C., Bocci G. (2017). The Key Role of Clinical and Community Health Nurses in Pharmacovigilance. Eur. J. Clin. Pharmacol..

[12] American Society of Hospital Pharmacy (1995). ASHP Guidelines on Adverse Drug Reaction Monitoring and Reporting. Am. J. Health Syst. Pharm..

[13] Swansea University ADRe (2018). ADRe—The Adverse Drug Reaction Profile: Helping to Monitor Medicines. https://www.swansea.ac.uk/adre/.

